# Combined Vitamin B12 and Folate Deficiency Presenting As Pseudo-Thrombotic Microangiopathy With a Disseminated Intravascular Coagulation (DIC)-Like Coagulation Profile: A Case Report

**DOI:** 10.7759/cureus.117118

**Published:** 2026-09-28

**Authors:** Moutaz W Sweileh, Hisham Asaad, Aya Abdi

**Affiliations:** 1 Department of Biomedical Sciences and Basic Clinical Skills, An-Najah National University, Nablus, PSE; 2 Department of Internal Medicine, An-Najah National University, Nablus, PSE; 3 Department of Gynecology, Rafidia Surgical Hospital, Nablus, PSE

**Keywords:** dic, folate deficiency, hematopathology, pseudo-thrombotic microangiopathy, vitamin b12 deficiency

## Abstract

Severe vitamin B12 deficiency can rarely present as pseudo-thrombotic microangiopathy (pseudo-TMA), characterized by anemia, thrombocytopenia, biochemical hemolysis, and schistocytosis, thereby mimicking thrombotic thrombocytopenic purpura (TTP). Concomitant abnormalities of conventional coagulation parameters may further complicate the presentation by raising concern for disseminated intravascular coagulation (DIC). We report an unusual presentation of severe combined vitamin B12 and folate deficiency with pseudo-TMA and a DIC-like coagulation profile.

A 56-year-old man with severe combined vitamin B12 and folate deficiency presented with profound pancytopenia, schistocytosis, biochemical hemolysis, inadequate reticulocytosis, and megaloblastic hematopoiesis despite normal mean corpuscular volume (MCV). Elevated D-dimer, prolonged prothrombin time (PT), and reduced fibrinogen produced a DIC-like profile. Hematologic and coagulation parameters improved within one week of starting vitamin B12 replacement; folic acid was initiated after that first week. Recognition of megaloblastic pseudo-TMA and its potential coagulation abnormalities may prevent misdiagnosis and unnecessary TTP-directed therapy.

## Introduction

Vitamin B12 (cobalamin) and folate are essential for DNA synthesis, and deficiencies are common causes of megaloblastic anemia resulting in ineffective hematopoiesis and intramedullary destruction of precursors, producing the characteristic megaloblastic alteration and, in severe cases, multilineage cytopenias or pancytopenia [1,2]. Dyserythropoiesis frequently leads to premature destruction of erythrocytes prior to their maturation and release into the peripheral blood; this manifests biochemically as elevated levels of lactate dehydrogenase (LDH) and total bilirubin (predominantly indirect bilirubin) alongside a diminished reticulocyte count (reticulocytopenia) [3].

Occasionally, severe vitamin B12 deficiency can produce a particularly challenging hematologic picture akin to thrombotic microangiopathy (TMA), characterized by anemia, thrombocytopenia, biochemical hemolysis, and the presence of red blood cell fragmentation or schistocytosis on the peripheral blood smear [4]. This constellation of features has thus been referred to as pseudo-TMA [5,6]. The clinical distinction from true TMA, particularly thrombotic thrombocytopenic purpura (TTP), is crucial because TTP requires urgent plasmapheresis, while megaloblastic pseudo-TMA responds to correction of the underlying vitamin deficiency. Markedly elevated LDH, an inappropriately low reticulocyte response, and the presence of cytopenias in other lineages may provide important clues favoring megaloblastic pseudo-TMA over TTP [7,8].

The diagnostic dilemma may become ever more challenging when abnormalities of conventional coagulation parameters coexist with the TMA-like hematologic picture. Although the coagulation profile is generally relatively preserved in TTP, thrombocytopenia and microangiopathic hemolysis accompanied by elevated fibrin degradation products, prolongation of coagulation times, and reduced fibrinogen levels may raise concern for disseminated intravascular coagulation (DIC) [9]. Such overlap has the potential to obscure the underlying megaloblastic process and broaden the differential diagnosis of an already unusual hematologic presentation.

We report the unusual coexistence of severe combined vitamin B12 and folate deficiency, pseudo-TMA features, and DIC-like coagulation abnormalities in a patient with profound pancytopenia and megaloblastic hematopoiesis. This case highlights the diagnostic overlap among megaloblastic pseudo-TMA, true TMA, and DIC and emphasizes the importance of integrating peripheral blood morphology, reticulocyte response, biochemical markers of hemolysis, coagulation studies, vitamin levels, and bone marrow findings.

## Case presentation

Clinical presentation

A 56-year-old man presented with dizziness and fatigue of months’ duration. He reported a regular, unrestricted diet, no medication use, and no gastrointestinal symptoms. He had no history of gastrointestinal surgery. There was no bleeding or symptoms suggestive of thrombosis. Neurological assessment was normal. Physical examination demonstrated pallor with no signs of jaundice, petechiae, hepatosplenomegaly, or lymphadenopathy. He was hemodynamically stable.

Initial hematological findings

Initial complete blood count (CBC) revealed profound pancytopenia, with a hemoglobin concentration of 4.0 g/dL, a white blood cell count of 1.48 x 10^9^/L, and a platelet count of 17 x 10^9^/L. The absolute neutrophil count was 1.09 x 10^9^/L. Despite the subsequent demonstration of severe vitamin B12 and folate deficiencies, the mean corpuscular volume (MCV) was within the reference range at 90 fL, with an elevated red cell distribution width of 18%. The remaining hematological parameters are summarized in Table 1.

**Table 1:** Laboratory findings at index presentation RDW: red cell distribution width; INR: international normalized ratio; aPTT: activated partial thromboplastin time; AST: aspartate aminotransferase; ALT: alanine aminotransferase

Parameter	Result	Unit	Reference range
Hematologic parameters			
White blood cell count	1.48	×10^9^/L	4-11
Hemoglobin	4.0	g/dL	14-18
Hematocrit	12.4	%	42-52
Red blood cell count	1.00	×10^12^/L	4-6
Mean corpuscular volume	90.0	fL	Laboratory range
RDW	18.0	%	Laboratory range
Platelet count	17	×10^9^/L	140-440
Absolute neutrophil count	1.09	×10^9^/L	Laboratory range
Reticulocyte parameters			
Reticulocyte count	1.35	%	0.5-1.5
Absolute reticulocyte count	38.9	×10^9^/L	23-70
Immature reticulocyte fraction	7.5	%	1.6-10.5
Reticulocyte hemoglobin equivalent	35.1	pg	32.1-38.8
Hemolysis-related parameters			
Lactate dehydrogenase	3136	U/L	Laboratory range
Total bilirubin	1.43	mg/dL	Laboratory range
Direct bilirubin	0.635	mg/dL	Laboratory range
Haptoglobin	1	mg/dL	30-200
Direct antiglobulin test	Negative	-	Negative
Coagulation parameters			
Prothrombin time	17.9	s	11-14
INR	1.42	-	0.8-1.2
aPTT	24.6	s	25-40
Fibrinogen	195	mg/dL	200-440
D-dimer	16.1	mg/L	0.1-0.4
Vitamin studies			
Vitamin B12	100	pg/mL	191-663
Folate	0.600	ng/mL	3.89-26.8
Other biochemical parameters			
AST	59.0	U/L	Laboratory range
ALT	32.2	U/L	Laboratory range
Albumin	3.82	g/dL	Laboratory range
Alkaline phosphatase	44.4	U/L	Laboratory range
Calcium	8.42	mg/dL	Laboratory range

Peripheral blood morphology

Peripheral blood smear examination showed marked anisopoikilocytosis with no macro-ovalocytes or hypersegmented neutrophils. Numerous red blood cell fragments were identified, morphologically consistent with true schistocytes (Figure 1). In conjunction with severe anemia and thrombocytopenia, the prominent red cell fragmentation initially raised concern for microangiopathic hemolysis.

**Figure 1:**
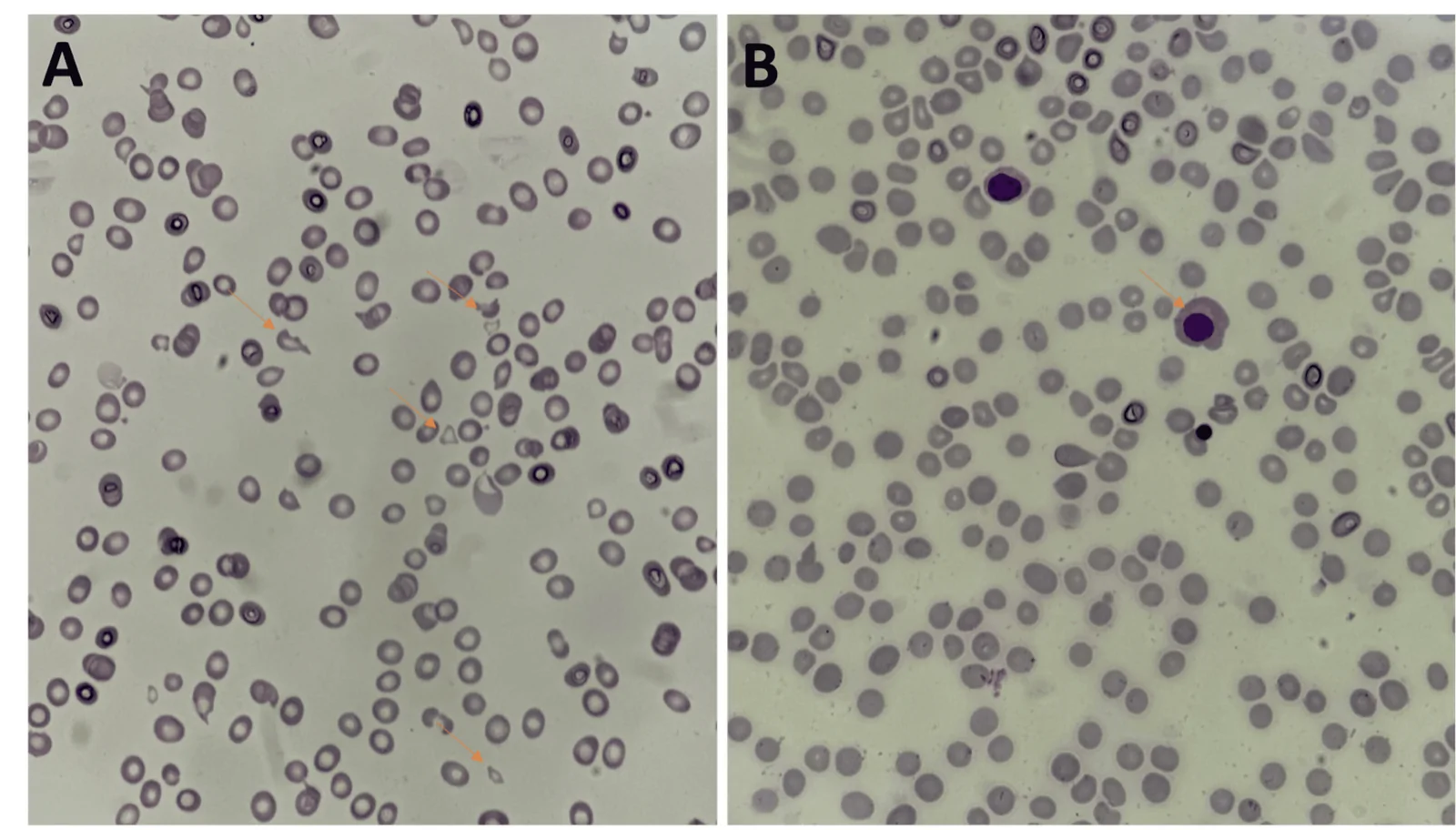
Peripheral blood smear findings (A-B) High-power view (40× objective) showing marked anisopoikilocytosis, including red blood cell fragments (schistocytes) and nucleated red blood cells, consistent with microangiopathic hemolysis (orange arrows).

Hemolysis and vitamin studies

Subsequent laboratory analysis revealed biochemical evidence of hemolysis, characterized by a markedly elevated LDH level of 3,136 U/L and a markedly reduced haptoglobin level of 1 mg/dL. Total and direct bilirubin levels were 1.43 mg/dL and 0.635 mg/dL, respectively, and the direct antiglobulin test was negative. Despite the biochemical evidence of hemolysis, the reticulocyte response was not increased, with a reticulocyte count of 1.35% and an absolute reticulocyte count of 38.9 x 10^9^/L. The reticulocyte count was measured one day after the initial CBC; a contemporaneous hematocrit was unavailable for calculation of a corrected reticulocyte count or production index. Vitamin studies demonstrated severe deficiencies of both vitamin B12 (100 pg/mL) and folate (0.6 ng/mL). Anti-intrinsic factor and anti-parietal cell antibody tests were not performed.

Coagulation findings

Coagulation studies demonstrated a prolonged prothrombin time (PT) of 17.9 seconds (international normalized ratio (INR), 1.42), with a markedly elevated D-dimer level of 16.1 mg/L and mildly reduced fibrinogen of 195 mg/dL. The activated partial thromboplastin time was 24.6 seconds. In conjunction with the severe thrombocytopenia, these findings raised concern for a DIC-like coagulation pattern. No formal DIC score was applied, and overt DIC was not established.

Evaluation for TMA

The combination of anemia, severe thrombocytopenia, biochemical hemolysis, and schistocytosis raised a differential diagnosis that included TMA. However, preserved renal function, absence of neurological manifestations, low reticulocyte response, pancytopenia, and severe B12/folate deficiency favored a megaloblastic process. ADAMTS13 activity was not measured.

Bone marrow findings

The bone marrow aspirate was hemodilute and suboptimal for assessment of overall marrow cellularity and lineage distribution. Nevertheless, the erythroid precursors present showed prominent megaloblastic and dyserythropoietic changes, including nuclear-cytoplasmic asynchrony, abnormal nuclear forms, and cytoplasmic granulation (Figure 2). The bone marrow trephine biopsy was 90% cellular and showed erythroid predominance with megaloblastic changes consistent with the aspirate findings (Figure 3). The overall bone marrow findings were consistent with megaloblastic hematopoiesis secondary to nutritional deficiency.

**Figure 2:**
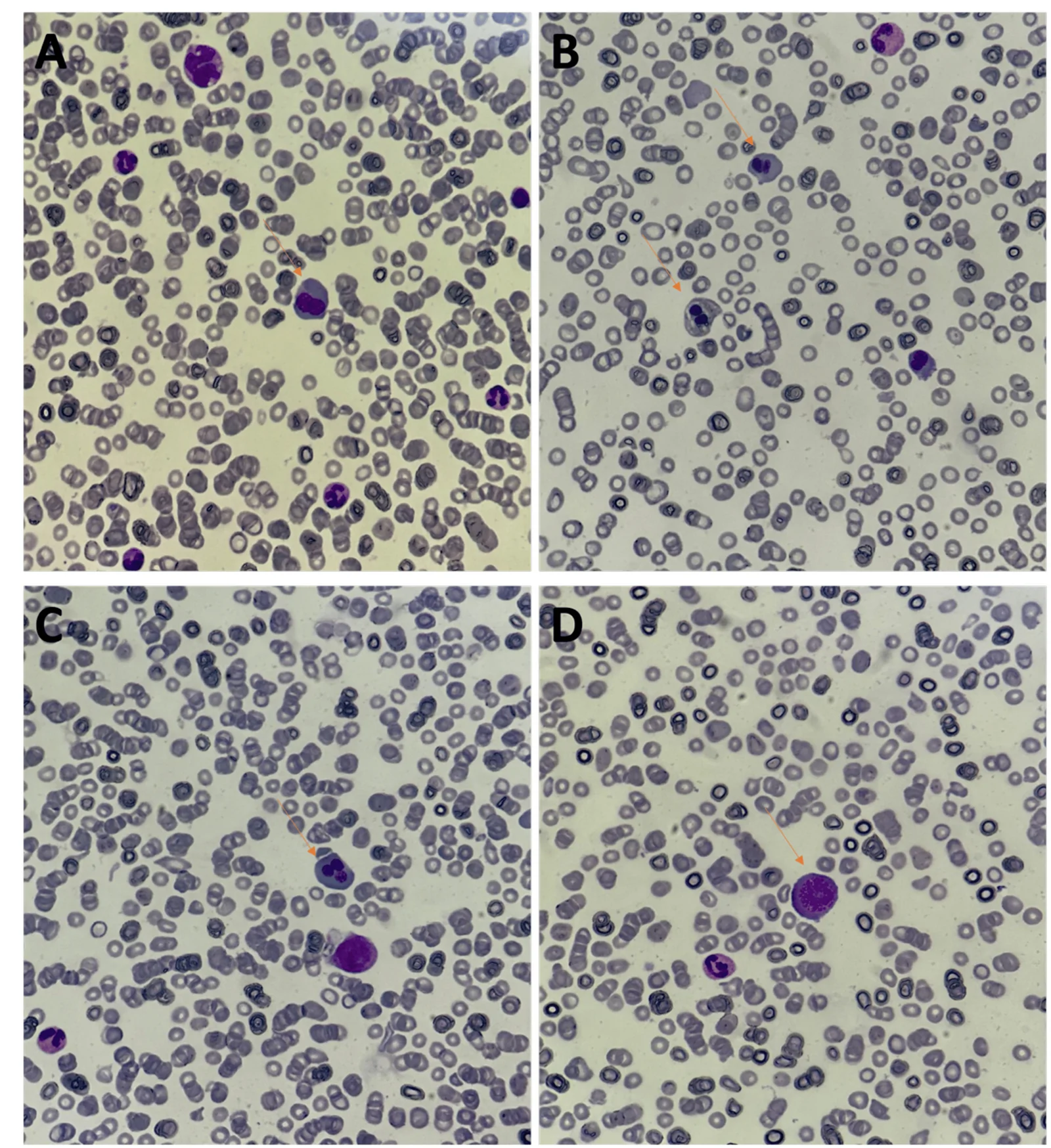
Bone marrow aspirate findings High-power view (40× objective) showing prominent dyserythropoietic changes (orange arrows): (A) nuclear budding; (B) irregular nuclear contours; (C) multinucleation; and (D) megaloblastic maturation with nuclear-cytoplasmic asynchrony.

**Figure 3:**
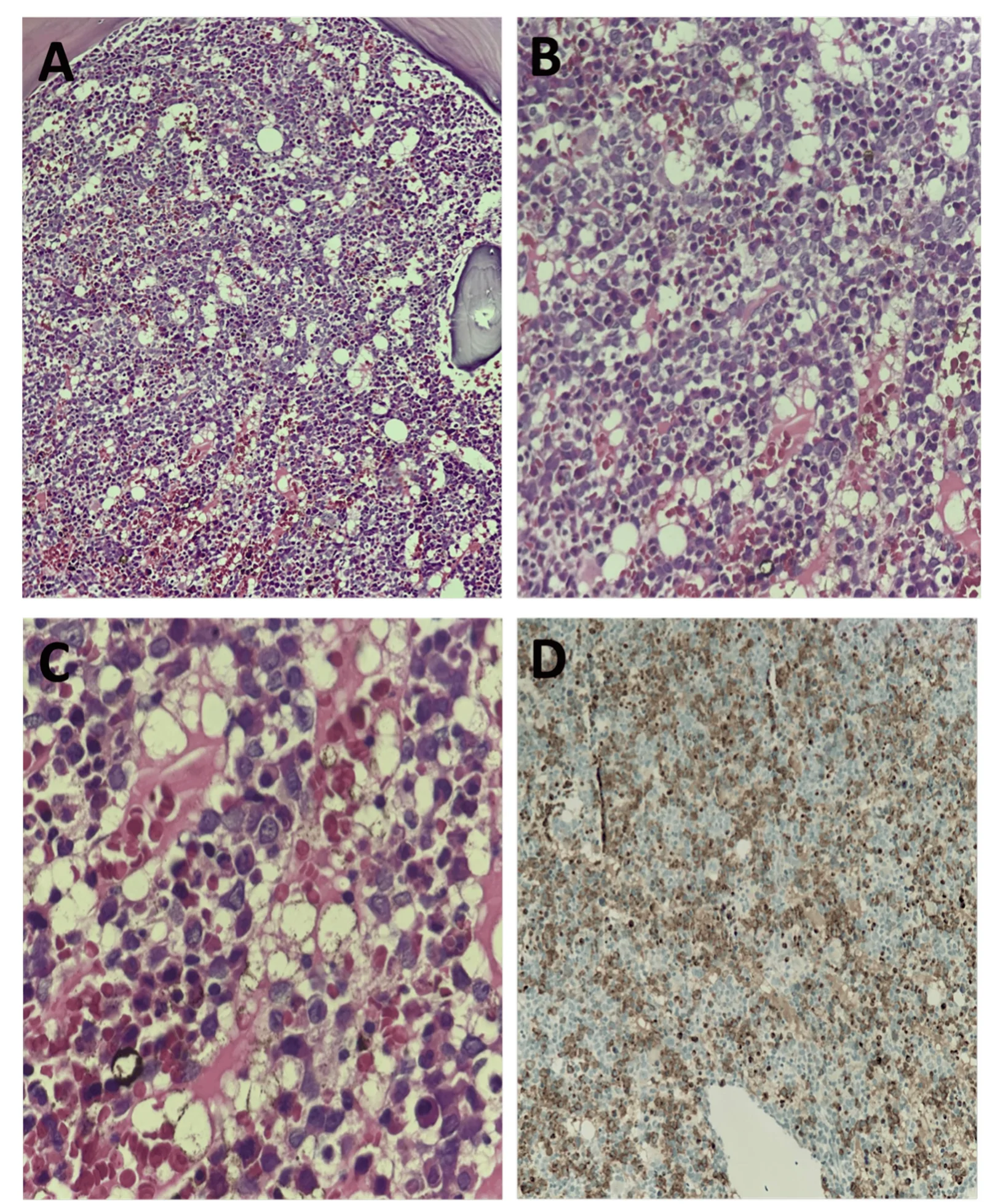
Bone marrow biopsy findings Bone marrow biopsy showing erythroid hyperplasia with left-shifted maturation at (A) low power, (B) medium power, and (C) high power views. (D) CD71 immunohistochemical stain highlights the expanded erythroid compartment and left-shifted erythroid maturation.

Treatment and follow-up

The patient received seven injections of vitamin B12, 1,000 µg (1 mg) per injection, administered every other day over two weeks. Oral folic acid, 5 mg once daily, was started one week after vitamin B12 treatment began. At the one-week follow-up, the patient demonstrated a marked hematologic response, with substantial improvement in anemia and leukopenia and complete recovery of the platelet count. A robust reticulocyte response was observed, accompanied by a marked decline in LDH and improvement in the coagulation profile. The detailed evolution of hematologic, hemolytic, and coagulation parameters is summarized in Table 2.

**Table 2:** Hematologic, hemolytic, and coagulation parameters following vitamin B12 and folate replacement Hb: hemoglobin; WBC: white blood cell count; ANC: absolute neutrophil count; MCV: mean corpuscular volume; LDH: lactate dehydrogenase; PT: prothrombin time; INR: international normalized ratio

Parameter	Admission	Week 1
Hb (g/dL)	4.0	9.3
WBC (×10^9^/L)	1.48	3.05
ANC (×10^9^/L)	1.09	2.09
Platelets (×10^9^/L)	17	366
MCV (fL)	90	93.7
Absolute reticulocytes (×10^9^/L)	38.9	155
LDH (U/L)	3,136	416
Total bilirubin (mg/dL)	1.43	1.34
Haptoglobin (mg/dL)	1	-
PT/INR	17.9/1.42	16.0/1.26
D-dimer (mg/L)	16.1	4.34
Fibrinogen (mg/dL)	195	364
Schistocytes	Present	-

## Discussion

Our patient presented with an unusual complex hematologic phenotype due to severe combined vitamin B12 and folate deficiency. Profound pancytopenia was accompanied by marked anisopoikilocytosis and numerous morphologically convincing schistocytes, together with biochemical evidence of hemolysis, including markedly elevated LDH and profoundly reduced haptoglobin. The reticulocyte response was abnormally low despite the severity of the anemia and biochemical hemolysis, suggesting inadequate erythropoiesis rather than an appropriate compensatory marrow response. This combination of hemolytic picture, thrombocytopenia, and schistocytosis is a recognized but uncommon presentation of severe megaloblastic anemia termed pseudo-TMA [8]. In our patient, bone marrow examination provided additional evidence of the underlying process, demonstrating hypercellular megaloblastic hematopoiesis with marked erythroid hyperplasia and nuclear-cytoplasmic asynchrony. The clinical picture was further complicated by a markedly elevated D-dimer, prolonged PT, and reduced fibrinogen, raising simultaneous concern for a consumptive coagulopathy. Thus, the initial presentation showed substantial phenotyping overlap among megaloblastic anemia, TMA, and DIC, illustrating the diagnostic difficulty that can arise when pseudo-TMA is accompanied by abnormalities of conventional coagulation profile.

Severe cobalamin and folate deficiency can produce a pseudo-microangiopathic phenotype through the profound disruption of erythropoiesis caused by impaired DNA synthesis. Nuclear maturation is delayed relative to cytoplasmic maturation, resulting in megaloblastic and ineffective erythropoiesis, with premature destruction of abnormal erythroid precursors in the bone marrow (intramedullary hemolysis) [1,2,10]. This intramedullary destruction accounts for the apparently paradoxical combination of biochemical hemolysis, particularly markedly elevated LDH and reduced haptoglobin, with an inappropriately low reticulocyte response, since much of the erythroid compartment is destroyed before mature erythrocytes can enter the circulation [3,4,7]. The mechanism underlying the accompanying peripheral red cell fragmentation is less clearly understood. Abnormally formed erythrocytes may exhibit modified membrane characteristics and diminished deformability, rendering them more prone to fragmentation within the microcirculation. Hyperhomocysteinemia caused by impaired cobalamin-dependent metabolism is proposed to provoke oxidative stress and cause endothelial cell injury, thereby resulting in erythrocyte damage and fragmentation [5,6]. These latter mechanisms, however, remain proposed explanations rather than firmly established mechanisms of schistocytosis in megaloblastic anemia. Importantly, schistocytes themselves are well documented in severe megaloblastic anemia and may coexist with marked anisopoikilocytosis [4]. This pathophysiologic pattern was particularly evident in our patient: despite severe anemia and biochemical hemolysis, with an LDH of 3,136 U/L and haptoglobin of 1mg/dL, the absolute reticulocyte count was only 38.9 x 10^9^/L; the peripheral smear demonstrated genuine red cell fragmentation within a background of marked anisopoikilocytosis, while bone marrow examination confirmed pronounced megaloblastic erythropoiesis.

The distinction between megaloblastic pseudo-TMA and true TTP is clinically crucial because both may present with severe anemia, thrombocytopenia, biochemical hemolysis, and schistocytosis, despite fundamentally different underlying mechanisms and management [5,7,8]. Several laboratory features may help distinguish the two entities. Compared with TTP, pseudo-TMA secondary to severe vitamin B12 deficiency is characterized by disproportionally elevated LDH and an inadequate reticulocyte response, reflecting extensive ineffective erythropoiesis and intramedullary precursor destruction rather than predominantly peripheral red cell destruction [5,7]. Concomitant leukopenia or pancytopenia further supports a megaloblastic process, whereas other hematopoietic lineages are generally preserved in TTP [5,7,8]. Macrocytosis may provide an additional clue but is not invariably present and therefore should not be required for recognition of severe vitamin deficiency [1,2]. Conversely, severe ADAMTS13 deficiency, defined as activity <10%, is characteristic of TTP and provides an important means of confirming the diagnosis when available [11]. Schistocytosis itself does not reliably distinguish the two conditions, since genuine schistocytes have been documented in megaloblastic anemia [4,5]. In our patient, hemoglobin of 4.0 g/dL, a platelet count of 17 ×10^9^/L, biochemical hemolysis, and prominent schistocytosis appropriately raised concern for TMA. However, the simultaneous leukopenia (1.48 ×10^9^/L), absolute reticulocyte count of only 38.9 ×10^9^/L despite profound anemia, severe combined vitamin B12 and folate deficiency, and unequivocal megaloblastic marrow strongly favored a pseudo-TMA phenotype related to ineffective hematopoiesis. A notable feature of this case is the normal MCV of 90 fL in the presence of profound vitamin B12 and folate deficiency. Because MCV is an average volume of red cells, it can mask variation in cell size; the elevated red cell distribution width (RDW) of 18% and significant anisopoikilocytosis in this patient support such variation. Coexisting microcytic processes may also mask macrocytosis; however, no specific contributing process was identified in this patient. Therefore, a normal MCV should not deter consideration of B12 and folate deficiency if other clinical and laboratory findings are suspicious for megaloblastic hematopoiesis.

The pronounced megaloblastic and dyserythropoietic marrow changes also raise a differential diagnosis of myelodysplastic neoplasm. However, severe vitamin B12 and folate deficiencies can themselves cause dysplastic-appearing marrow changes. In this patient, the documented deficiencies, low reticulocyte response at presentation, and marked hematologic improvement following vitamin replacement favor deficiency-related ineffective hematopoiesis.

An additional diagnostic dilemma in our patient was the presence of significant coagulation irregularities alongside the pseudo-TMA phenotype. Unlike TTP, where microvascular thrombosis is mainly driven by platelet-rich von Willebrand factor thrombi and standard coagulation parameters remain largely intact, DIC is marked by widespread coagulation activation leading to fibrin generation, depletion of platelets and coagulation factors, and subsequent fibrinolysis, usually resulting in heightened D-dimer levels, extended coagulation times, and, in severe instances, diminished fibrinogen [9,11]. Our patient demonstrated several features suggestive of this latter process, including a markedly elevated D-dimer of 16.1 mg/L, prolonged PT of 17.9 seconds (INR 1.42), severe thrombocytopenia, and reduced fibrinogen of 195 mg/dL, despite the absence of an identifiable alternative clinical trigger for DIC. Similar DIC-like coagulation abnormalities have occasionally been described in severe vitamin B12 deficiency, suggesting that profound megaloblastic disease may, in rare circumstances, be accompanied by activation of the coagulation system [9]. The mechanism remains unclear. Suggested rationales include endothelial cell injury linked to hyperhomocysteinemia and coagulation activation arising from significant ineffective hematopoiesis and cellular degradation; nonetheless, a direct causal relationship remains unproven, and homocysteine levels were not assessed in our patient [6,12]. Thus, these abnormalities are best described as a DIC-like laboratory pattern observed alongside severe megaloblastic anemia, rather than confirmed DIC. Their improvement after vitamin replacement shows a temporal association but does not establish causation; the absence of an identifiable alternative DIC trigger does not prove that vitamin deficiency caused them.

Most reported cases of megaloblastic pseudo-TMA have been attributed to severe vitamin B12 deficiency, whereas folate deficiency as an isolated cause appears to be considerably less common [5,13]. Published B12-deficient cases consistently describe varying combinations of severe anemia, thrombocytopenia, schistocytosis, markedly elevated LDH, and an inadequate reticulocyte response, with pancytopenia and megaloblastic marrow findings providing additional evidence for ineffective hematopoiesis rather than primary TMA [5,8,14]. Folate deficiency can produce a similar phenotype: isolated folate-deficiency pseudo-TMA has been reported with pancytopenia, schistocytosis, biochemical hemolysis, and megaloblastic erythroid hyperplasia, followed by hematologic improvement after folate replacement [13]. Our patient differs in having profound deficiencies of both vitamins simultaneously, with vitamin B12 of 100 pg/mL and folate of 0.6 ng/mL; therefore, the hematologic phenotype cannot confidently be attributed to either deficiency in isolation. More importantly, the combination of profound pancytopenia, genuine schistocytosis, an inadequate reticulocyte response, megaloblastic marrow morphology, and a prominent DIC-like coagulation profile makes the presentation particularly instructive. Severe B12 deficiency accompanied by overt or DIC-like coagulation abnormalities has been described previously [9], but such cases appear uncommon relative to the broader pseudo-TMA literature. Accordingly, the value of the present case lies not in claiming a unique association, but in demonstrating the convergence of severe combined B12/folate deficiency, pseudo-TMA morphology, ineffective hematopoiesis, and significant coagulation abnormalities within a single clinicopathologic presentation.

Recognition of megaloblastic pseudo-TMA carries significant therapeutic consequences, as suspected TTP necessitates immediate disease-targeted intervention, such as therapeutic plasma exchange and corticosteroids, while pseudo-TMA resulting from vitamin deficiency is addressed through the correction of the deficiency. Failure to recognize this differentiation could consequently subject individuals with megaloblastic anemia to unnecessary plasma exchange and additional TTP-directed therapies [5,7,8]. The response to vitamin supplementation may offer significant retrospective validation for the diagnosis, as the reestablishment of effective erythropoiesis triggers an initial reticulocyte response, subsequently leading to the recovery of peripheral cytopenias and the normalization of biochemical indicators of intramedullary hemolysis. The patient exhibited a remarkable response within one week of starting vitamin B12 replacement, before folic acid was initiated: the absolute reticulocyte count increased from 38.9 to 155 ×10^9^/L, platelet count from 17 to 366 ×10^9^/L, and hemoglobin from 4.0 to 9.3 g/dL, whilst LDH decreased from 3,136 to 416 U/L. Notably, the coagulation abnormalities improved in parallel, as D-dimer decreased from 16.1 to 4.34 mg/L, fibrinogen increased from 195 to 364 mg/dL, and PT/INR improved from 17.9 s/1.42 to 16.0 s/1.26. The hematologic response supports vitamin deficiency as the cause of the ineffective erythropoiesis. The coagulation parameters improved over the same period, but this parallel change does not establish that vitamin deficiency caused the coagulation abnormalities.

The etiology of the combined vitamin deficiencies was not established. Although the patient reported an unrestricted diet, no medication use, no gastrointestinal symptoms, and no gastrointestinal surgery, testing for autoimmune gastritis was not performed. An underlying absorption disorder therefore cannot be excluded.

## Conclusions

Severe B12 and folate deficiency should be considered in patients presenting with pancytopenia, biochemical hemolysis, and schistocytosis, even in the absence of macrocytosis. An inappropriately low reticulocyte response despite severe anemia, together with markedly elevated LDH and cytopenias involving multiple lineages, may provide important clues to ineffective hematopoiesis and help distinguish megaloblastic pseudo-TMA from true TTP. Bone marrow examination, when clinically indicated, can further support the diagnosis by demonstrating characteristic megaloblastic hematopoiesis. As seen in our patient, abnormalities of conventional coagulation parameters may further complicate the presentation by producing a DIC-like phenotype and should not obscure an underlying megaloblastic process. Recognition of this diagnostic overlap is important because vitamin-deficiency pseudo-TMA responds to vitamin replacement, whereas TTP requires urgent TTP-directed therapy. Careful integration of peripheral blood morphology, reticulocyte response, hemolysis markers, coagulation studies, vitamin levels, and, when available, bone marrow findings can facilitate the correct diagnosis and prevent unnecessary treatment.
